# Seroprevalence of Toscana virus in blood donors in mainland Portugal

**DOI:** 10.1186/s13071-025-06726-x

**Published:** 2025-03-03

**Authors:** Rafael Rocha, Elif Kurum, Nazli Ayhan, Rémi Charrel, Carla Maia

**Affiliations:** 1 Global Health and Tropical Medicine (GHTM), Associate Laboratory in Translation and Innovation Towards Global Health, LA-REAL, Instituto de Higiene e Medicina Tropical (IHMT), Universidade Nova de Lisboa (UNL), Lisbon, Portugal; 2 Unité Des Virus Émergents (UVE: Aix-Marseille Univ, Università di Corsica, IRD 190, Inserm 1207, IRBA), Marseille, France; 3 National Reference Center for Arboviruses, Inserm-IRBA, Marseille, France

**Keywords:** *Phlebovirus*, Toscana virus, Seroprevalence, Blood donors, Portugal

## Abstract

**Background:**

Toscana virus (TOSV; *Phlebovirus toscanaense*), a phlebovirus transmitted by sand flies, is a growing public health concern in the Mediterranean region, with infections often being asymptomatic but potentially leading to neuroinvasive disease. Despite its presence in neighboring countries, data on TOSV seroprevalence in Portugal are limited. This study aimed to estimate the national seroprevalence of TOSV among blood donors in mainland Portugal and explore associations with sociodemographic factors and *Leishmania* infection.

**Methods:**

A cross-sectional study was conducted using serum samples from 3593 blood donors across mainland Portugal, collected between February and June 2022. Anti-TOSV antibodies were detected via microneutralization assay, and anti-*Leishmania* antibodies had previously been tested using ELISA. Sociodemographic data were obtained from self-administered questionnaires. Seroprevalence was estimated by region, and multivariate logistic regression was used to identify factors associated with TOSV infection.

**Results:**

Overall, the estimated national true seroprevalence of TOSV was 2.6% (95% CI 2.1–3.1%). Regional seroprevalence varied significantly, with the highest values (up to 14.8%) in Alto Alentejo, Baixo Alentejo, Douro, Alto Tâmega e Barroso and Oeste regions. Multivariate analysis showed that age ≥ 50 years (aOR 1.70, 95% CI 1.04–2.77), residing in the Alentejo region (aOR 3.05, 95% CI 1.85–5.02) and positive/borderline *Leishmania* serology (aOR 2.31, 95% CI 1.29–4.15) were significantly associated with TOSV infection.

**Conclusions:**

This study highlights new areas of TOSV circulation in Portugal, particularly in regions with higher *Leishmania* seroprevalence and visceral leishmaniasis incidence, suggesting co-circulation of these pathogens. Although a lower seroprevalence was obtained compared to neighboring countries, TOSV should still be considered in the differential diagnosis of viral meningitis and encephalitis in Portugal, especially in potentially high-risk regions. Further research is needed to better understand the ecological drivers of TOSV distribution in Portugal.

**Graphical Abstract:**

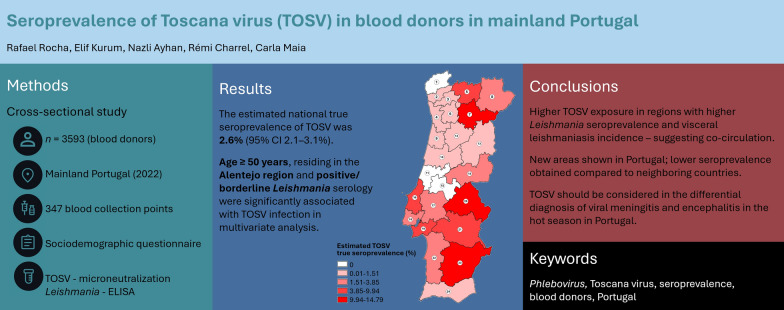

**Supplementary Information:**

The online version contains supplementary material available at 10.1186/s13071-025-06726-x.

## Background

The *Phlebovirus* genus, belonging to the Phenuiviridae family, includes 68 species [according to the latest classification of the International Committee on Taxonomy of Viruses [1], some of which are pathogenic to humans. These viruses are transmitted by arthropod vectors, including phlebotomine sand flies, ticks [2] and mosquitoes (e.g. Rift Valley fever virus, RVFV [3]). Species of this genus that are transmitted by sand flies have been particularly neglected in terms of professional and public awareness, research funding, surveillance and public health prioritization, owing, in part, to their typically mild or asymptomatic clinical presentation. However, they represent a potential threat to human and animal health as there is increasing potential for their geographic expansion in the context of climate change and globalization.

Phleboviruses transmitted by sand flies in the Old World with well-known pathogenic potential (in humans) are currently grouped into three species, all of which are circulating in the Mediterranean region: Naples virus (*Phlebovirus napoliense*), Sicilian virus (*Phlebovirus siciliaense)* and Toscana virus (TOSV; *Phlebovirus toscanaense*) [4]. Infection by these viruses can result in a broad clinical spectrum, although asymptomatic infection seems to be the most common outcome (only detectable through seropositivity) [5]. In symptomatic individuals, the most frequent presentation, known as sandfly fever or Pappataci fever, usually manifests as a nonspecific, self-limited febrile syndrome (with an average duration of 3 days), often characterized by headaches, muscle pain and nausea/vomiting [6]. However, TOSV is neurotropic, and infection of the central nervous system (CNS) can result in meningitis, encephalitis and other less common forms of involvement (such as peripheral neuropathy and Guillain-Barré syndrome). Fatal cases or those with permanent sequelae are rare [7].

Advancement in the understanding of the epidemiology of TOSV has primarily been realized through molecular biology and/or serological studies in cerebrospinal fluid (CSF) and serum from patients with suspected acute CNS infection (prospectively and retrospectively). The percentage of CNS infections attributed to TOSV in these studies was variable: 4.6–6.5% in Italy [8, 9] (although this percentage reached 81.0% in specific regions and years during the summer [10]), 10.0% in Tunisia [11] and 1.0% in Portugal [12]. Sporadic cases of TOSV CNS infection acquired in Portugal have also been documented previously, including in foreign tourists in 1985 [13] and in 1996 [14] and in Portuguese inhabitants (a total of 11 cases, diagnosed between 2002 and 2008, reported in two studies [15, 16]). Additionally, the recent identification of neuroinvasive disease caused by TOSV in individuals (without travel history) from areas previously considered non-endemic, such as southwest Germany, is noteworthy [17].

In addition, serological cross-sectional studies of human and animal populations in confirmed or potentially endemic areas have also significantly contributed to epidemiological knowledge. Some of these studies have targeted blood donors in the Mediterranean Basin, showing TOSV seroprevalences ranging from 0 to 26% in France, Italy and Spain [18–22]. In Portugal, three seroprevalence studies on TOSV in healthy individuals showed values of 1.1–5.3% in the Porto [23], Lisbon, Santarém [20] and Setúbal districts [24, 25]. Additionally, in Portugal, recent seroprevalence rates of 3.7–6.8% for TOSV were detected in dogs and cats in various regions [26–28]. In wild animals, exposure to TOSV has been detected in foxes and wolves but not in rodents [29].

*Phlebotomus perniciosus* has been identified as the main vector of TOSV [30]. This species seems to be widespread in Portugal, according to data from national sand fly surveillances, gradually implemented since 2016 [31]. *Phlebotomus perniciosus* has also been recognized as the main vector of *Leishmania infantum* in the western Mediterranean [32], and cocirculation of the two pathogens has been suggested by the detection in sand flies collected in the same endemic areas, including in Portugal [33], and statistical association in seroprevalence studies in humans [34]. Other sand fly species have been detected in Portugal (*Phlebotomus ariasi, P. papatasi, P. sergenti* and *Sergentomyia minuta*) but seem to have a more localized distribution in the country [31].

In this context, this study aimed to estimate the national seroprevalence of TOSV in blood donors in mainland Portugal through the detection of antibodies using the microneutralization technique and to study the association between the presence of anti-TOSV antibodies and anti-*Leishmania* antibodies as well as various sociodemographic factors in this population and the practices of individuals regarding daily activities and pet ownership.

## Methods

### Study population

Data and samples for this work had previously been collected for another cross-sectional *Leishmania* seroprevalence study [35]. A detailed description of the study methodology can be found in [35], and a summary is provided in Supplementary Table S1. That study targeted the population of people who donate blood in mainland Portugal through the Portuguese Institute of Blood and Transplantation (IPST) or the immunohemotherapy departments (IHDs) of public hospitals in the Alentejo and Algarve regions. The IPST and the IHDs perform regular blood collections in fixed centers as well as collections in shifting stations in rural and urban areas. In 2021, over 190,000 blood donations were performed in these institutions [36, 37], after a strict and standardized triage process. Mainland Portugal is located in Southwest Europe, bordering Spain and the Atlantic Ocean, and is divided into seven NUTS2 regions, 24 NUTS3 regions [37], 278 municipalities and 2882 parishes. According to the 2021 national census, the population of mainland Portugal aged 15–64 years old was 6,257,752 inhabitants [38].

### Data and sample collection

The sampling was stratified by municipality. The individuals enrolled in the original study presented to one of the institutions collaborating in the study from February to June 2022 and were considered fit for blood donation. Only individuals aged 18–65 years old were included. Participant enrollment was performed in non-randomly selected blood collection sessions, but, in each session, invitation to participate in the study was random (by time of presentation at the blood collection center/station). Each participant filled in a self-administered structured paper questionnaire about sociodemographic aspects; 1.5 ml of the routinely collected serum was sent to the Instituto de Higiene e Medicina Tropical (IHMT) and stored at – 20 ºC for the study. Only participants who consented to be included in further studies were included in the TOSV seroprevalence study.

Categorical variables extracted from the questionnaire were analyzed mostly using the original categories provided as answer options, but regrouping was performed in some cases. Classification of professions was performed using the ESCO Classification of Occupations, developed by The European Commission in 2010 [39]. Classification of parishes as rural or non-rural followed the Portuguese Rural Development Program 2014–2020 [40]. TOSV endemic travel destinations included countries where human or animal infection/disease has been reported, according to [7].

### Serological study

Anti-TOSV antibodies were detected by microneutralization technique. Human sera were heat-inactivated at 56 °C for 30 min, then diluted from 1:10 to 1:80 and mixed in equal volumes with 1000 TCID₅₀ of TOSV (MRS2014–44725) in 96-well plates. Following a 1-h incubation at 37 °C, 100 μl of a Vero E6 cell suspension (5 × 10^5^ cells/ml) was added. Each plate included positive and negative controls. After 5 days, cytopathic effect (CPE) was assessed, and neutralization titers were determined by TCID50. Detection of antibodies in any titer was considered a positive result. A sensitivity of 98.1% and specificity of 98.8% were assumed for this technique; these were calculated for CPE-based VNT with reference to PRNT90 for Zika virus [41] since data for TOSV are not available, but the same technique was used.

Antileishmanial antibody detection in each serum sample had already been performed using ELISA (*Leishmania* ELISA IgG + IgM, Vircell®, Spain), following the manufacturer’s instructions and cut-offs. These kits simultaneously detect IgM and/or IgG antibodies against *Leishmania*; the plate’s wells are coated with an unspecified *L. infantum* antigen. The sensitivity and specificity of the ELISA, according to the manufacturer, are 97% and 99%, respectively. A single determination was performed for each serum sample. Samples were classified as positive, negative or borderline (when optical density was < 10% lower or higher than the average value of the borderline controls).

### Statistical analysis

True prevalence was estimated at national and regional level based on the following formula: true prevalence (TP) = (test prevalence—1 + specificity)/(sensitivity—1 + specificity). The corresponding 95% CIs were obtained using the Wilson’s method on Epitools© Epidemiological Calculators [42, 43].

Absolute and relative frequencies and hypothesis testing were performed using IBM® SPSS® Statistics Version 29.0. Geographical representation and analysis of results were obtained using QGIS® Version 3.22. Descriptive statistics were expressed as absolute frequencies and percentages for categorical variables and as medians with interquartile intervals (IQIs) for asymmetric continuous variables (e.g. age). Missing or unknown data were excluded from denominators, unless stated otherwise. Comparisons between groups were performed using Pearson Chi-square test for categorical variables (or Fisher’s exact in case of failure of the assumptions of the χ2 test). A value of *p* < 0.05 was considered statistically significant.

Multivariate analysis was conducted to identify sociodemographic factors associated with TOSV infection. This analysis was performed through a multiple binary logistic regression model, analyzing variables with statistical meaning in the univariate analysis (*p* < 0.20) and some biologically relevant or potentially confounding variables.

For those variables that remained significant, crude odds ratio (OR) were updated to adjusted odds ratio (aOR) with 95% CI. The Hosmer-Lemeshow test was used for assessing goodness of fit in each multiple logistic regression model [44]. The reference categories used for each independent variable are specified in each multivariate analysis results table.

## Results

In total, 3593 participants were included in this study. Participants were recruited in 636 blood collection sessions in 347 different collection sites. Globally, 235 of the 278 municipalities of mainland Portugal were represented. Missing municipalities were concentrated in the eastern Algarve, Alto Alentejo, Coimbra and Alto Minho NUTS3 regions. Median age was 41 years old, and it was higher in Península de Setúbal, Alentejo and Algarve NUTS2 regions (Table 1). Participants were evenly distributed between sexes globally and in most regions, except for the Alentejo and the Algarve, where male sex was clearly predominant. Differences in education level, occupation, contact with domestic animals, ownership of dogs and practice of outdoor activities during nighttime between NUTS2 regions were noted and are presented in Table 1.

**Table 1:** Sociodemographic characteristics of the participants, globally and by NUTS (Nomenclature of Territorial Units for Statistics) 2 region

	Global	Norte	Centro	OVT	GL	PS	Alentejo	Algarve
Total	100 (3593/3593)	33.6 (1206/3593)	14.7 (527/3593)	8.0 (289/3593)	19.6 (703/3593)	7.6 (273/3593)	10.0 (359/3593)	6.6 (236/3593)
Median age (years) (IQR)	41 (31–48)	39 (30–47)	40 (29–47)	41 (29–49)	41 (30–50)	44 (33–49)	43 (35–50)	42 (35–50)
Male sex (%)	49.8 (1787/3585)	47.4 (570/1203)	47.1 (248/526)	50.9 (147/289)	48.9 (343/701)	44.7 (122/273)	61.8 (222/359)	57.7 (135/234)
Education level^a^								
Basic (1–4)	1.7 (60/3503)	1.5 (18/1166)	1,4 (7/514)	3.1 (9/286)	1.0 (7/688)	1.1 (3/267)	3.4 (12/351)	1.7 (4/231)
Basic (5–9)	16.5 (579/3503)	20.0 (233/1166)	16.3 (84/514)	18.2 (52/286)	9.4 (65/688)	13.9 (37/267)	19.7 (69/351)	16.9 (39/231)
Secondary (10–12)	44.0 (1542/3503)	43.3 (505/1166)	42.4 (218/514)	49.3 (141/286)	39.5 (272/688)	40.1 (107/267)	51.3 (180/351)	51.5 (119/231)
Bachelor's	26.1 (914/3503)	23.6 (275/1166)	28.8 (148/514)	22.7 (65/286)	32.3 (222/688)	32.2 (86/267)	19.9 (70/351)	20.8 (48/231)
MSc/PhD	11.6 (408/3503)	11.6 (135/1166)	11.1 (57/514)	6.6 (19/286)	17.7 (122/688)	12.7 (34/267)	5.7 (20/351)	9.1 (21/231)
Occupation^b^								
Student	10.0 (287/2882)	9.2 (89/965)	13.6 (59/435)	12.4 (30/241)	13.0 (72/554)	5.9 (13/221)	5.7 (16/279)	4.3 (8/187)
Unemployed	3.4 (99/2882)	4.5 (43/965)	2.8 (12/435)	3.3 (8/241)	3.6 (20/554)	2.7 (6/221)	1.4 (4/279)	3.2 (6/187)
Retired	1.7 (50/2882)	1.0 (10/965)	0.7 (3/435)	1.7 (4/241)	2.9 (16/554)	1.4 (3/221)	3.2 (9/279)	2.7 (5/187)
Armed forces (0)	1.9 (54/2882)	1.3 (13/965)	1.6 (7/435)	3.3 (8/241)	1.1 (6/554)	2.7 (6/221)	2.5 (7/279)	3.7 (7/187)
Managers, professionals and technicians (1–3)	39.4 (1135/2882)	37.2 (359/965)	37.7 (164/435)	31.5 (76/241)	50.0 (277/554)	49.3 (109/221)	33.0 (92/279)	31.0 (58/187)
Clerical support, service and sales (4–5)	25.7 (741/2882)	24.4 (235/965)	22.5 (98/435)	26.1 (63/241)	19.9 (110/554)	24.0 (53/221)	35.1 (98/279)	44.9 (84/187)
Agriculture, craft, industry and elementary (6–9)	17.9 (516/2882)	22.4 (216/965)	21.1 (92/435)	21.6 (52/241)	9.6 (53/554)	14.0 (31/221)	19.0 (53/279)	10.2 (19/187)
Others								
Regular contact with domestic animals	70.9 (2427/3424)	70.5 (803/1139)	79.4 (397/500)	74.9 (209/279)	61.7 (418/677)	68.8 (181/263)	74.4 (253/340)	73.5 (166/226)
Practice of outdoor activities during nighttime	24.6 (803/3268)	19.9 (218/1093)	29.2 (138/473)	26.9 (71/264)	23.0 (149/648)	23.6 (60/254)	33.2 (108/325)	28.0 (59/211)
Ownership of dog(s)	48.3 (1706/3530)	46.7 (549/1175)	55.9 (289/517)	58.9 (169/287)	36.9 (257/696)	46.7 (127/272)	56.3 (197/350)	50.6 (118/233)
Positive or borderline *Leishmania* serology	7.8 (281/3593)	9.5 (115/1206)	10.2 (54/527)	8.7 (25/289)	5.3 (37/703)	5.9 (16/273)	4.5 (16/359)	7.6 (18/236)

^a^Numbers in brackets refer to number of years completed of formal school education

^b^Numbers in brackets refer to the numbers of the categories in the classification of European Skills, Competences and Occupations

GL, Grande Lisboa; IQR, interquartile range; MSc, Master of Science; OVT, Oeste e Vale do Tejo; PhD, Doctor of Philosophy; PS, Península de Setúbal

### Serological results

**Fig. 1:**
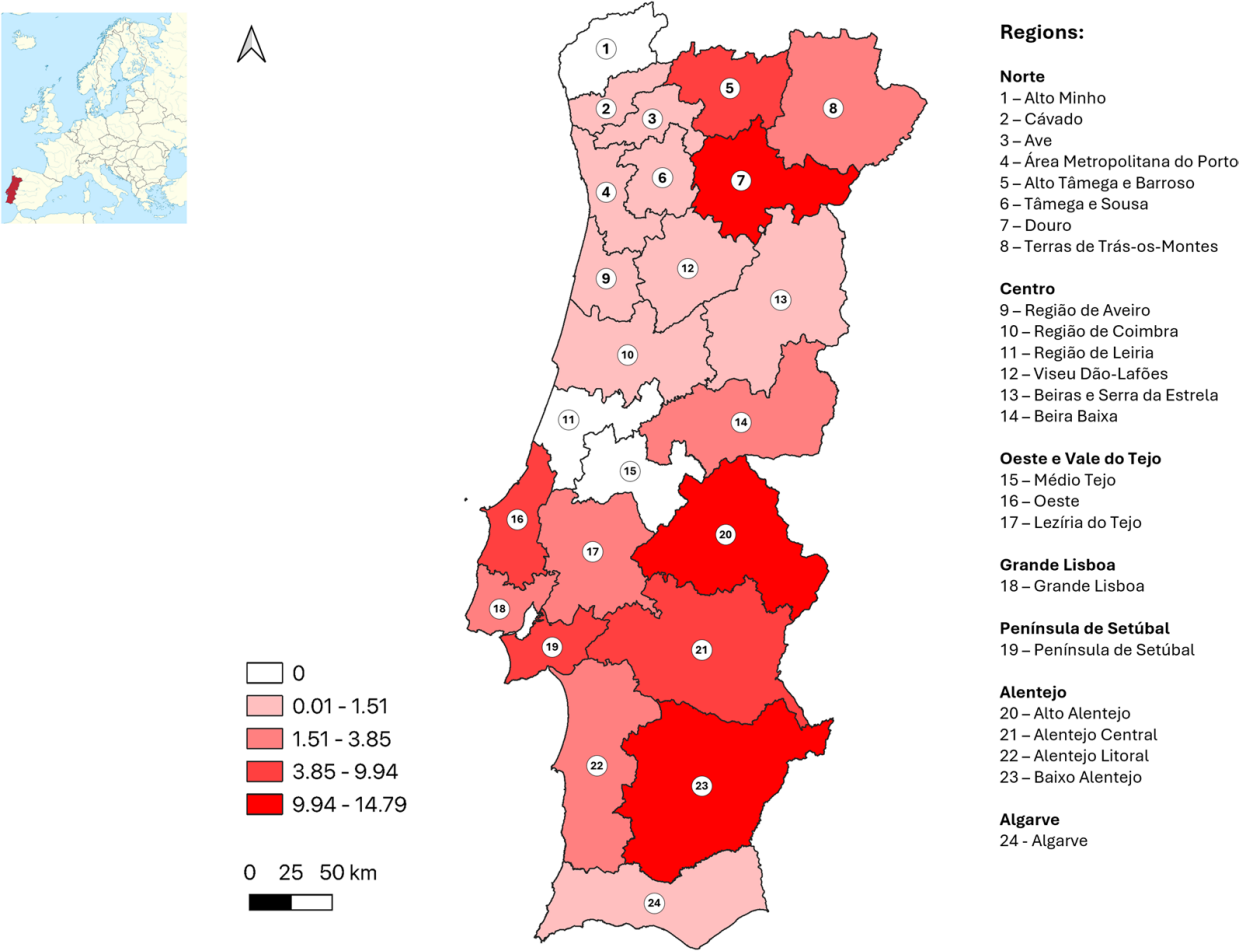
Distribution of estimated TOSV true seroprevalence values (%) by NUTS3 region

In total, 142 (4.0%) samples were positive for TOSV antibodies. Distribution of positive results by NUTS2 and three regions is given in Table 2. Adjusted positivity rates were considered slight deviations between the expected and the achieved sample size by municipality. Figure 1 shows the distribution of true seroprevalence estimates by NUTS3 region.

**Table 2:** Distribution of positive results by NUTS (Nomenclature of Territorial Units for Statistics) 2 and 3 region and estimated true prevalence

Region	Sampling sites (*n*)	Samples (*n*)	Positive samples (*n*)	Crude positivity rate (%)	Adjusted positivity rate (%)	True prevalence (%)	95% Confidence interval
Norte	149	1206	37	3.1	2.9	1.8	1.1–2.6
Alto Minho	12	68	0	0.0	0.0	0.0	0.0–5.3
Cávado	17	148	3	2.0	1.8	0.6	0.1–3.7
Ave	16	144	3	2.1	2.1	0.9	0.1–4.0
Área Metropolitana do Porto	60	558	15	2.7	2.6	1.5	0.7–2.8
Alto Tâmega e Barroso	4	29	3	10.3	9.3	8.3	2.9–21.8
Tâmega e Sousa	23	152	3	2.0	1.9	0.8	0.1–3.9
Douro	13	75	9	12.0	14.1	13.3	7.4–22.8
Terras de Trás-os-Montes	4	32	1	3.1	3.7	2.6	0.5–13.5
Centro	91	527	9	1.7	1.7	0.5	0.2–1.6
Região de Aveiro	19	134	2	1.5	2.0	0.8	0.1–4.2
Região de Coimbra	21	108	2	1.9	1.5	0.3	0.1–3.3
Região de Leiria	14	99	0	0.0	0.0	0.0	0.0–3.7
Viseu Dão-Lafões	15	65	2	3.1	2.3	1.2	0.3–7.7
Beira Baixa	7	40	2	5.0	4.0	2.9	0.5–13.2
Beiras e Serra da Estrela	15	81	1	1.2	1.8	0.6	0.1–4.5
Oeste e Vale do Tejo	38	289	16	5.5	5.4	4.3	2.5–7.3
Oeste	12	139	12	8.6	7.8	6.8	3.5–12.1
Médio Tejo	16	64	1	1.6	0.9	0.0	0.0–5.7
Lezíria do Tejo	10	86	3	3.5	3.5	2.4	0.6–8.1
Grande Lisboa	16	703	31	4.4	4.4	3.3	2.1–4.9
Península de Setúbal	9	273	14	5.1	5.1	4.0	2.2–7.1
Alentejo	42	360	30	8.3	8.5	7.5	5.2–10.7
Alentejo Litoral	10	70	3	4.3	4.0	2.9	0.8–9.8
Baixo Alentejo	8	100	12	12.0	11.4	10.6	6.0–18.0
Alto Alentejo	9	56	7	12.5	15.5	14.8	7.5–26.1
Alentejo Central	15	134	8	6.0	5.7	4.6	2.1–9.6
Algarve	2	236	5	2.1	2.7	1.5	0.6–3.9
Total	347	3593	142	4.0	3.7	2.6	2.1–3.1

Global estimated true seroprevalence was 2.6% (95% CI 2.1–3.1%). At the NUTS3 level, values ranged from 0.0 to 14.8%, with highest seroprevalences in the Alto Alentejo, Douro and Baixo Alentejo regions. There was a statistically significant difference in the proportion of positive results between NUTS2 regions (*p* < 0.001, Chi-square test, χ2 = 33.2, df = 6).

**Fig. 2:**
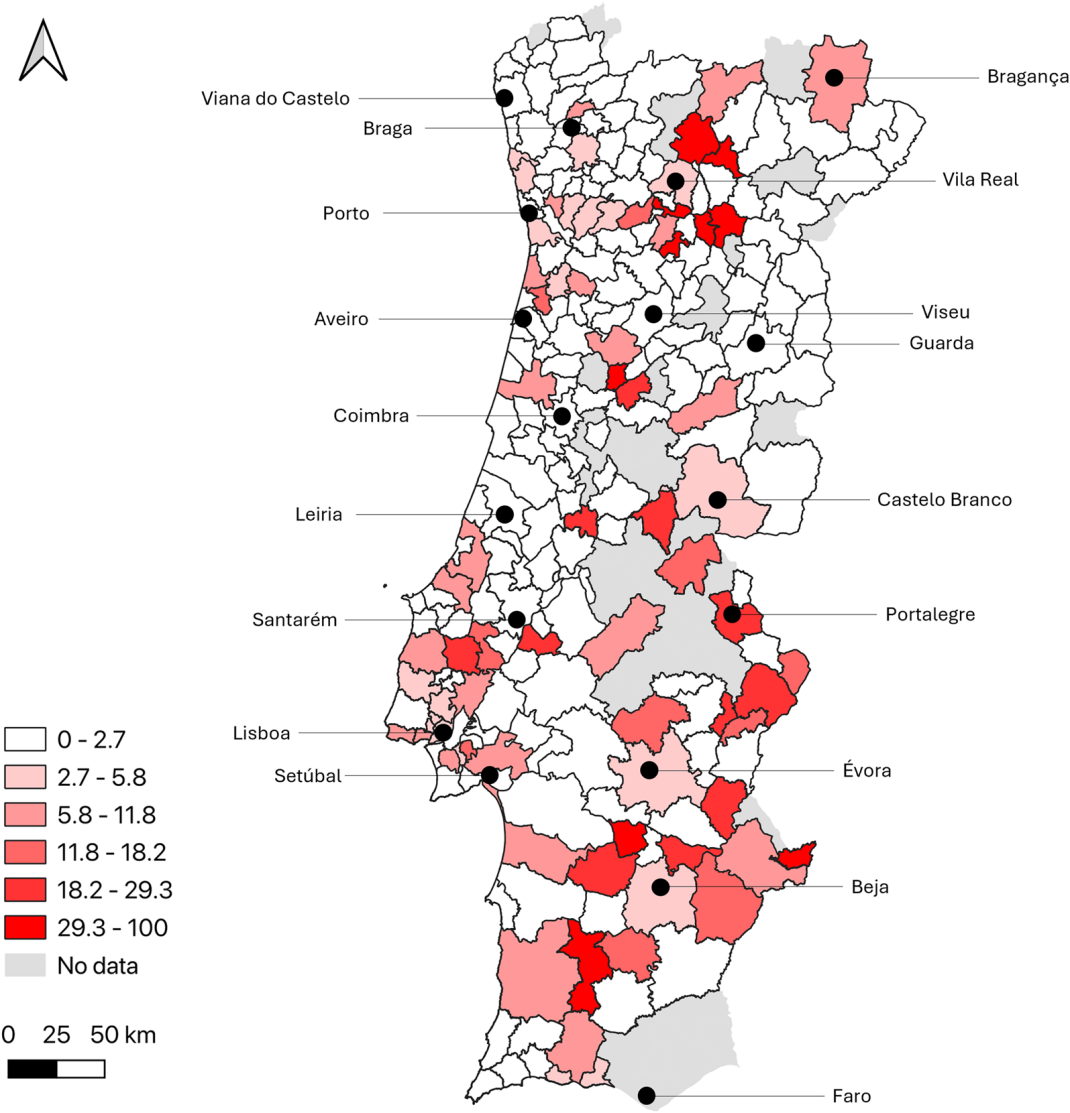
Percentage of TOSV-positive samples by municipality (natural breaks were used to define categories; the approximate location of district capital cities is highlighted)

**Fig. 3:**
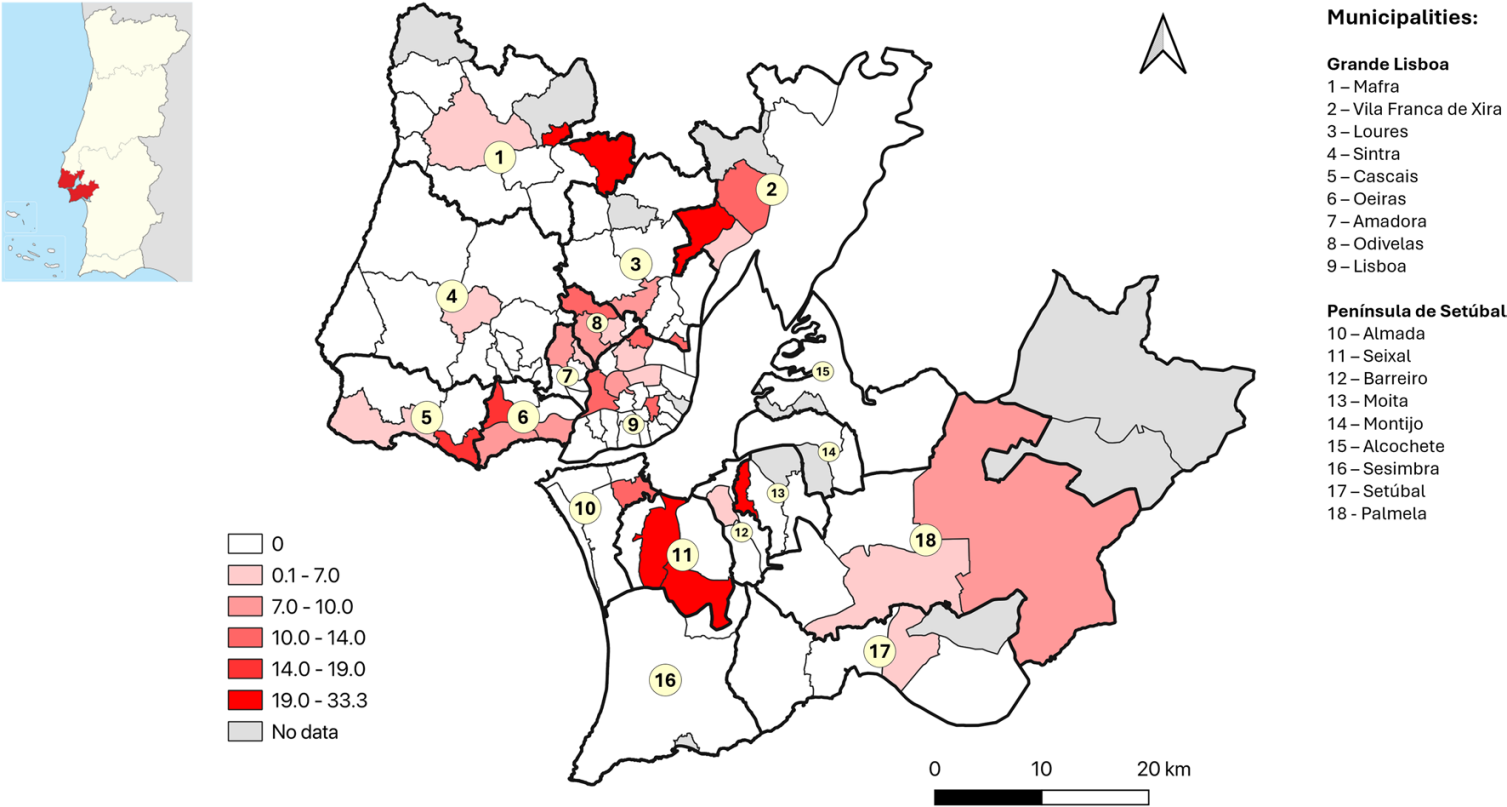
Percentage of positive samples in each parish of the Grande Lisboa and Península de Setúbal regions

To allow a more detailed analysis of the geographical distribution of possible exposure to TOSV, the percentage of positive samples was calculated by municipality and is given in Fig. 2. Municipalities where at least 15 samples were collected (*n* = 73) with the highest percentages (> 9.0%) were (by descending order): Alenquer, Moita, Serpa, Alcobaça, Seixal and Caldas da Rainha. Additionally, to understand possible clustering of positive cases at a more local level, percentage by parish is illustrated for the Grande Lisboa and Península de Setúbal NUTS3 regions in Fig. 3.

### Associations between sociodemographic variables and asymptomatic infection

In univariate analysis, residing in the Alentejo region (considering the Centro as a reference category), age > 44 years old and basic level of education were significantly associated with a positive serological result (Table 3). People with a positive result more often reported regular practice of outdoor activities during nighttime (*p* = 0.056, Chi-square test, χ2 = 3.6, df = 1) and more often had a positive or borderline *Leishmania* serology (*p* = 0.060, Chi-square test, χ2 = 3.5, df = 1), although these associations did not reach statistical significance. Residence in a rural parish, regular contact with domestic animals and ownership of dogs were not significantly associated with a positive serology. In multivariate analysis, factors associated with a positive TOSV serological result were age ≥ 50 years old (aOR 1.70, 95% CI 1.04–2.77, *p* = 0.033), residing in the Alentejo region (aOR 3.05, 95% CI 1.85–5.02, *p* < 0.001) and having a positive or borderline *Leishmania* serology (OR 2.31, 95% CI 1.29–4.15, *p* = 0.005) (Table 4).

**Table 3:** Distribution of participants by serological result and by category for sociodemographic variables

Variables	Categories	Result of TOSV serology, % (n)		*p* value
		Positive	Negative	
Sex	Male	53.9 (76/141)	49.7 (1711/3444)	0.326 (χ2 = 0.96, df = 1)
	Female	46.1 (65/141)	50.3 (1733/3444)	
Age (years)	18–24	13.6 (19/140)	12.9 (435/3364)	0.049* (χ2 = 9.5, df = 4)
	25–34	14.3 (20/140)	20.4 (685/3364)	
	35–44	23.6 (33/140)	28.9 (972/3364)	
	45–54	31.4 (44/140)	26.9 (905/3364)	
	55–65	17.1 (24/140)	10.9 (367/3364)	
Level of education^a^	1–4	5.1 (7/138)	1.6 (53/3365)	0.004* (χ2 = 15.6, df = 4)
	5–9	22.5 (31/138)	16.3 (548/3365)	
	10–12	43.5 (60/138)	44.0 (1482/3365)	
	Bachelor's	21.0 (29/138)	26.3 (885/3365)	
	MSc/PhD	7.8 (11/138)	11.8 (397/3365)	
Occupation^b^	Student	8.3 (9/108)	10.0 (278/2774)	
	Retired	4.6 (5/108)	1.6 (45/2774)	
	Unemployed	3.7 (4/108)	3.4 (95/2774)	
	0	1.1 (1/90)	2.2 (53/2356)	0.199 (χ2 = 4.6, df = 3)
	1–3	36.7 (33/90)	46.8 (1102/2356)	
	4–5	37.8 (34/90)	30.0 (707/2356)	
	6–9	24.4 (22/90)	21.0 (494/2356)	
Travel abroad to endemic country^c^ (< 2 years previously)	Yes	13.1 (18/137)	15.1 (499/3294)	0.519 (χ2 = 0.42, df = 1)
	No	86.9 (119/137)	84.9 (2795/3294)	
Type of parish of residence	Non-rural	53.5 (76/142)	58.3 (2008/3447)	0.263 (χ2 = 1.3, df = 1)
	Rural	46.5 (66/142)	41.7 (1439/3447)	
Regular contact with domestic animals	Yes	73.1 (98/134)	70.8 (2329/3290)	0.558 (χ2 = 0.34, df = 1)
	No	26.9 (36/134)	29.2 (961/3290)	
Regular contact with wild animals	Yes	4.6 (6/130)	4.2 (132/3117)	0.833 (χ2 = 0.04, df = 1)
	No	95.4 (124/130)	95.8 (2985/3117)	
Practice of outdoor activities during nighttime	Yes	31.7 (40/126)	24.3 (763/3142)	0.056 (χ2 = 3.6, df = 1)
	No	68.3 (86/126)	75.7 (2379/3142)	
Use of nets in windows/doors	Yes (all/some)	28.1 (38/135)	23.7 (780/3289)	0.236 (χ2 = 1.4, df = 1)
	None	71.9 (97/135)	76.3 (2509/3289)	
Ownership of dog(s)	Yes	52.9 (74/140)	48.0 (1628/3390)	0.262 (χ2 = 1.26, df = 1)
	No	47.1 (66/140)	52.0 (1762/3390)	
Positive or borderline *Leishmania* serology	Yes	12.0 (17/142)	7.6 (264/3451)	0.060 (χ2 = 3.5, df = 1)
	No	88.0 (125/142)	92.4 (3187/3451)	
NUTS2 region of residence	Norte	26.1 (37/142)	33.9 (1169/3451)	< 0.001* (χ2 = 33.2, df = 6)
	Centro	6.3 (9/142)	15.0 (518/3451)	
	Oeste e Vale do Tejo	11.3 (16/142)	7.9 (273/3451)	
	Grande Lisboa	21.8 (31/142)	19.5 (672/3451)	
	Península de Setúbal	9.9 (14/142)	7.5 (259/3451)	
	Alentejo	21.1 (30/142)	9.5 (329/3451)	
	Algarve	3.5 (5/142)	6.7 (231/3451)	

^a^Categories refer to number of years of formal school education completed

^b^Category numbers refer to the numbers of the categories in the classification of European Skills, Competences and Occupations

^c^Algeria, Bosnia and Herzegovina, Bulgaria, Croatia, Cyprus, France, Greece, Italy, Kosovo, Malta, Morocco, Spain, Tunisia, Turkey

MSc, Master of Science; NUTS, Nomenclature of Territorial Units for Statistics; PhD, Doctor of Philosophy; TOSV, Toscana virus

^*^Statistically significant

**Table 4:** Potential risk factors for TOSV infection according to logistic regression models to estimate crude and adjusted odds ratio values

Potential risk factor	Univariate			Multivariate		
	% in Sample	Crude OR	95% CI	Adjusted OR	95% CI	*p*-value
Age ≥ 50 years old	21.3	1.93	1.35–2.77	1.70	1.04–2.77	0.033*
Male sex	49.8	1.18	0.84–1.66	1.14	0.74–1.76	0.542
Residing in Alentejo	10.0	2.54	1.67–3.86	3.05	1.85–5.02	< 0.001*
Basic education level	18.2	1.74	1.19–2.57	1.61	0.95–2.73	0.074
Outdoor activities at night	24.6	1.45	0.99–2.13	1.45	0.92–2.29	0.108
Retired or profession 4–9^a^	45.4	1.59	1.08–2.35	1.26	0.80–1.97	0.320
Pos/Bd *Leishmania* serology	7.8	1.64	0.97–2.77	2.31	1.29–4.15	0.005*
Constant				0.018		< 0.001
Hosmer-Lemeshow test				Sig. = 0.192		

^a^Clerical support workers; service and sales workers; skilled agricultural, forestry and fishery workers; craft and related trades workers; plant and machine operators and assemblers; elementary occupations

CI, confidence interval; OR, odds ratio; Pos/bd, positive/borderline

^*^Statistically significant

## Discussion

This study represents the first nationwide TOSV human seroprevalence study in Portugal. National true seroprevalence was estimated at 2.6%, with regional values ranging from 0.0 to 14.8%. A comparison with previous regional studies shows overlapping positivity rates in the Península de Setúbal (5.1 vs. 5.3%; samples collected in 2019) [25] and Área Metropolitana do Porto (2.7 vs. 3.9%; published in 2011) [23] NUTS3 regions. Notably, the same serological technique was used in the first case, while ELISA was used in the second. The national and regional seroprevalence estimates of the present study are, however, considerably lower than suggested in regions of other Mediterranean countries (using the same serological technique), including in neighboring Spain (26.3% in the Granada province) [19] and especially in North Africa: Algeria (46.2%) [45] and Tunisia (41.0%) [46]. Factors explaining the lower seroprevalence in Portugal could be related to aspects of the presence, abundance and infection rate of the main vector of TOSV (*Phlebotomus perniciosus*) in the country. Although *P. perniciosus* seems to be widespread in Portugal [31], its abundance has not been well characterized. Data from national surveillance (2016–2023) show presence of *P. perniciosus* in all/most of the municipalities sampled in the Baixo Alentejo, Alto Alentejo and Douro [31], which were regions with higher estimated TOSV seroprevalence in the present study. Of note, no sand fly collection was systematically performed in regions such as Área Metropolitana do Porto, Cávado and Alto Tâmega e Barroso [31], nor have entomological data from these regions been reported previously in the literature, reflecting the insufficient knowledge on sand fly distribution in the northern part of Portugal. Additionally, TOSV has, to date, only been detected in sand flies collected in the Algarve region [in one of 45 pools distributed across the country [47]. Other sand fly species detected in Portugal (*P. ariasi*, *P. papatasi*, *P. sergenti* and *Sergentomyia minuta*) could also be competent for TOSV maintenance and transmission [48], but they seem to have a more localized distribution in the country [31]. Most of northern and coastal Portugal presents a Warm Summer Mediterranean (Csb) climate [49], where higher humidity and lower summer temperatures could be less favorable for sand flies [50], hence potentially contributing to a generally lower TOSV seroprevalence in these regions (such as Alto Minho, Aveiro and Leiria) compared to regions with a Hot Summer Mediterranean (Csa) climate. Lastly, differences in human behavior and exposure to sand fly bites could also play a role.

In this study, a likely intense exposure to TOSV is shown in areas where it had not previously been documented, namely in the interior of the Norte and in the Alentejo NUTS2 regions. Interestingly, several NUTS3 regions where a higher estimated seroprevalence was found are the same ones where a higher incidence of visceral leishmaniasis (VL) was documented in Portugal between 2010 and 2020, including the Alto Alentejo, Baixo Alentejo and Alto Tâmega e Barroso but not the Algarve [51]. In the Algarve, however, caution is required when interpreting the results, given the limited geographic coverage, as the lack of data from specific municipalities may have impacted the overall findings. In contrast, other regions where a higher estimated TOSV seroprevalence was calculated presented a lower VL incidence, such as the Oeste region. In this region, however, when analysis at municipality level is performed, the areas with positive TOSV donors overlap the areas where VL cases and *Leishmania*-positive donors were detected, namely Alenquer, Caldas da Rainha and Alcobaça (seropositivity in the first two exceeded 9% for both TOSV and *Leishmania*) [35]. Taken together, these findings support the co-circulation of TOSV and *Leishmania* in several regions of mainland Portugal. Additional studies could provide further insights into the environmental and sociodemographic factors associated with the differential exposure to these sand fly-borne pathogens in Portugal. Although information on the presence of different sand fly species at a nation-wide level has been increasing in recent years [31], data concerning relative abundance are still scarce and restricted to a few regions (e.g. the Algarve [52]).

The present study showed a significant association between detection at the individual level of TOSV and *Leishmania* antibodies in healthy individuals in an endemic region. This association was already reported [19, 34] and is likely explained by exposure to the common main vector in Portugal, *P. perniciosus*. Other factors associated with detection of TOSV antibodies in the present study have also been suggested in previous studies in blood donors, namely older age [19, 53], which could be related to the cumulative risk of exposure in endemic regions.

Male sex was not significantly associated with seropositivity in the present study; a similar finding was reported in a previous study in Corsica [54] but not in another study in Turkey [53]. This discrepancy may be attributed to variations in exposure factors and prevalence of high-risk behaviors by sex, which may differ across regions and contexts. Notably, TOSV symptomatic infections are consistently observed more frequently in males than in females [55], suggesting that other factors such as sex-related immune response and viral susceptibility may be relevant for symptomatic presentation.

Other factors found not to be significant in this study, in contrast with previous studies, included residing in rural areas or regular contact with domestic animals [53, 56]. Of note, the natural reservoir of TOSV still needs to be determined [57, 58].

In summary, these findings and the previous studies suggest that, in Portugal, raised clinical awareness regarding TOSV should be promoted, especially in the Alto Alentejo, Baixo Alentejo, Douro and Alto Tâmega e Barroso regions. TOSV should be considered in the differential diagnosis of suspected viral meningitis and encephalitis, especially from May to October, considering knowledge on the seasonal activity of phlebotomine sand flies in Portugal [59]. This clinical approach will also rely on the availability of molecular biology and serology techniques to detect TOSV in highly endemic areas.

Using serological techniques to determine exposure to TOSV has some limitations, including possible cross-reactivity with antigenically related phleboviruses. However, neutralization-based assays are considered the gold standard for the confirmation of *Phlebovirus* antibody specificity [60]. Seroneutralization is the most stringent and specific assay, in particular for Toscana virus [46].

Calculating sensitivity and specificity for microneutralization assays presents several challenges due to the absence of a definitive gold standard to determine true infection status, low prevalence rates, potential cross-reactivity and the selection of appropriate thresholds. In this study, we utilized sensitivity and specificity values derived from Zika virus data as these metrics were not available for the TOSV microneutralization technique. Since the Zika virus microneutralization assays were conducted using the same methodology and within the same laboratory unit, these values serve as a reasonable proxy. However, differences between the two viruses and their specific immune responses may introduce a slight bias in the results. Despite the many caveats for the use of serology for individual determination of previous TOSV infection status, the detection of antibodies can be relevant from a public health perspective, especially when comparing the findings between different regions and by crossing the results with distribution of human cases and evidence from TOSV in mammals and vectors following a One Health approach. This knowledge could be helpful to control human TOSV infections, highlighting regions where environmental interventions to reduce phlebotomine breeding sites and increased adherence to the use of arthropod repellent (from dusk to dawn) could be recommended. Future research should focus on understanding the specific ecological niches of TOSV in Portugal.

These findings cannot be transposed to the general Portuguese mainland population, since only healthy people aged 18–65 years were included and the profile of people who donate blood could be different from the age-matched general population in each region and between regions. In addition, the representativeness even of the blood donor population itself could have been affected by the difficulty in obtaining a truly probabilistic sample because of logistic constraints in some regions.

## Conclusions

To our knowledge, this study provides the first nationwide estimate of TOSV seroprevalence in mainland Portugal, revealing a true seroprevalence of 2.6%, with significant regional variation. These findings suggest an increased exposure to TOSV in previously undocumented areas, particularly in parts of the Norte and Alentejo regions, and reinforce the association between TOSV and *Leishmania* infections. While the study's design focused on blood donors, the results underscore the importance of raising clinical awareness of TOSV, particularly in regions with higher seroprevalence and during the active sand fly season. Future research should explore the ecological factors contributing to the regional distribution of TOSV as well as potential public health interventions to reduce exposure to this emerging pathogen following a One Health approach.

## Supplementary Information

Additional file 1.

## Data Availability

The datasets generated and analyzed during the current study are not publicly available due to confidentiality commitment with the participants, as stated in the consent declaration, but are available from the corresponding author on reasonable request.
