# Supplementary material for: Seroprevalence of Toscana virus in blood donors in mainland Portugal
Source: Parasit Vectors. 2025 Mar 3;18:82. doi: 10.1186/s13071-025-06726-x (PMC11874794; doi:10.1186/s13071-025-06726-x)
Supplement: Supplementary file 1 — Additional file 1. [file 13071_2025_6726_MOESM1_ESM.docx]

**Supplementary Information**

[**Additional file 1: Supplementary Table S1**](https://static-content.springer.com/esm/art%3A10.1186%2Fs13071-024-06640-8/MediaObjects/13071_2024_6640_MOESM1_ESM.docx)

Summary of the methodology of the previous study - cross-sectional study on the prevalence of anti-*Leishmania* antibodies in blood donors in Mainland Portugal (1). Samples obtained for that study (stored at a temperature of -20ºC) were used for the present study.

| **Population, materials, and methods** | |
| --- | --- |
| **Study population** | This cross-sectional national study was carried out in blood donors in mainland Portugal, which is in Southwest Europe, bordering Spain and the Atlantic Ocean. According to the 2021 national census, the population of mainland Portugal aged 15 to 64 years was 6,257,752 inhabitants (2). To ensure a nationwide coverage of sampling, this study was performed in collaboration with the Portuguese Institute of Blood and Transplantation (IPST) and with the immunohemotherapy departments (IHDs) of five hospital centers in the Alentejo and Algarve regions. The IPST and the IHDs perform regular blood collections in fixed centers as well as in shifting stations in rural and urban areas. In 2021, over 190,000 blood donations were performed in these institutions. Individuals are considered eligible for donation after a strict triage conducted by a trained health professional, to exclude acute disease and several chronic conditions and risk behaviors. Additionally, capillary hemoglobin levels are determined; men with less than 13.5 g/dl and women with less than 12.5 g/dl are automatically excluded from donating. |
| **Sample size calculation** | Sample size was estimated using the Epitools^©^ Epidemiological Calculators (3,4). At least 3200 individuals were needed to estimate a 95% confidence interval (CI) for prevalence, considering an expected maximum global (national) seroprevalence of 9% (based on Spanish regional studies (5,6,7) and a minimum sensitivity and specificity of the serological test used of 85% and 90%, respectively, and considering a desired precision of 0.02 to a 95% CI. Additionally, this sample size would allow the detection of small differences in seroprevalence between NUTS3 regions, with a power of 95%, using a Chi-square test.  Sampling was stratified by municipality: the number of participants enrolled from each municipality was proportional to the fraction of the mainland population (aged 15–64 years) living in that region, according to the most recent census data and assuming a similar distribution for blood donors. For five NUTS3 regions in southern Portugal (Alto Alentejo, Alentejo Central, Baixo Alentejo, Alentejo Litoral, and Algarve), where higher seroprevalence was expected a priori (according to human incidence data derived from VL cases reported to the National Surveillance System) (8), recruitment of additional participants was planned in order to increase the precision of estimates, increasing the total sample size to 3494. |
| **Eligibility criteria** | Individuals enrolled in this study presented to one of the institutions collaborating in the study from February to June 2022 and were considered fit for blood donation. Only individuals aged 18 to 65 years were included. Blood donation must have been completed, including collection of a serum sample for routine serological testing. |
| **Data and sample collection** | Participant enrollment was performed in non-randomly selected blood collection sessions, to ensure a maximum number of municipalities were surveyed. In some municipalities, more than one session was required to complete the calculated sample sizes, and, in this case, different zones of the municipality were preferably surveyed. Blood donation sessions exclusive to specific professional groups (such as police officers or firefighters) were generally avoided. A fixed number of participants was set for each session (1 to 8). Blood donors were randomly invited to the study, according to the hour of presentation at the blood collection center/station–considering non-consecutive, pre-defined time slots. This procedure differed in one center (in the Lisbon Metropolitan Area), due to logistic reasons, where all donors in each session were invited consecutively, by order of arrival, until the sample size for the municipality was fulfilled.  Recruitment was performed in both fixed donation centers and mobile stations, except in the Algarve region. The participants were informed about the study and signed an informed consent declaration. Each participant completed a self-administered structured paper questionnaire about sociodemographic aspects. This questionnaire was pretested in a convenience sample of 40 blood donors from the Norte and Área Metropolitana de Lisboa regions. 1.5 ml of serum taken from the peripheral blood sample collected for routine serological testing was sent to the Instituto de Higiene e Medicina Tropical (IHMT) and stored at − 20 ºC for the study. |
| **Serological study** | Antileishmanial antibody detection in each serum sample was performed using enzyme-linked immunosorbent assay (ELISA) (*Leishmania* ELISA IgG+IgM, Vircell^®^, Spain), following the manufacturer’s instructions and cut-offs. These kits simultaneously detect immunoglobulin M (IgM) and/or IgG antibodies against *Leishmania*; the wells of the plate are coated with an unspecified *L. infantum* antigen. The sensitivity and specificity of the ELISA, according to the manufacturer, are 97% and 99%, respectively. A single determination was performed for each serum sample. Samples were classified as positive, negative, or borderline (when optical density was less than 10% lower or higher than the average value of the borderline controls). Participants from whom positive samples were taken were considered to have been exposed to *Leishmania*—either past or current asymptomatic infection. |

**References**

1. Rocha, R., Gonçalves, L., Conceição, C. *et al.* Prevalence of asymptomatic *Leishmania* infection and knowledge, perceptions, and practices in blood donors in mainland Portugal. *Parasites Vectors* **16**, 357 (2023). https://doi.org/10.1186/s13071-023-05980-1
2. INE. Resultados Provisórios–Censos 2021. Resultados Provisórios. 2021.
3. Sargent E. Epitools Epidemiological Calculators. Ausvet. 2018. <https://epitools.ausvet.com.au/>. Accessed 22 Feb 2023
4. Humphry RW, Cameron A, Gunn GJ. A practical approach to calculate sample size for herd prevalence surveys. Prev Vet Med. 2004;65:173–88.
5. Aliaga L, Ceballos J, Sampedro A, Cobo F, López-Nevot MÁ, Merino-Espinosa G, et al. Asymptomatic Leishmania infection in blood donors from the Southern of Spain. Infection. 2019;47:739–47.
6. Pérez-Cutillas P, Goyena E, Chitimia L, De la Rúa P, Bernal LJ, Fisa R, et al. Spatial distribution of human asymptomatic *Leishmania infantum* infection in southeast Spain: a study of environmental, demographic and social risk factors. Acta Trop. 2015;146:127–34.
7. Martín-Sánchez J, Rodríguez-Granger J, Morillas-Márquez F, Merino-Espinosa G, Sampedro A, Aliaga L, et al. Leishmaniasis due to *Leishmania infantum*: integration of human, animal and environmental data through a one health approach. Transbound Emerg Dis. 2020;67:2423–34.
8. SPMS. Doenças de Declaração Obrigatória . Portal da Transparência. 2018. <https://transparencia.sns.gov.pt/explore/dataset/doencas-de-declaracao-obrigatoria/table/?disjunctive.regiao_de_saude_de_notificacao&disjunctive.residencia_nut_iii&disjunctive.doenca_de_declaracao_obrigatoria&disjunctive.sexo&disjunctive.grupo_etario&sort>. Accessed 22 Feb 2023
